# Poisoned by Mercury from a Tooth Filling

**Published:** 1872-01

**Authors:** 


Poisoned by Mercury from a Tooth Filling.—The following, from a Nebraska paper, shows that the "amalgam question" receives some attention in the West: "Last Wednesday evening the intelligence was noised about that Mr. John C. Smith, a middle-aged man, unmarried, who lived in a small house next west of the residence of S. W. Allen, was dead. It was known in town that he had been suffering for some days with a swelled face and neck, coming from a tooth, which Dr. Keef, of Marysville, had lately filled, but his death was not thought possible. Dr. Sprague attended the deceased at first, but afterwards called Drs. Davis and Buffon, all of whom agreed that he was suffering from the effects of mercury, present in the amalgam used in filling one of his teeth. The filling had salivated the unfortunate man, and as the inside of his mouth, throat and windpipe swelled, respiration was hindered, and it finally ceased altogether.

"Poultices were applied, and other means used to reduce the swelling, but all to no purpose. Mr. Smith died about 7 o'clock Wednesday evening.

"By request of a number of the citizens, Coroner Buchanan next day ordered a *post mortem* examination to be made of the remains and an inquest to be held. Dr. Davis made the examination, opening the chest and taking out the lungs, and also extracting the filled tooth. No signs of any other disease were found except that caused by the mercury, and it was made clear to the jury by the Doctor that this caused the death.

"The jury was composed of the following persons: L. Y. Coffin, C. F. Buchanan, Joseph Saunders, James Charles, W. J. Dunn, O. M. Enlow.

"S. W. Allen testified that Mr. S. had asked him to see a dentist for him; that Mr. S. afterwards told him that he had been to see Dr. Keef, who had filled the tooth; that he had filled it without killing the nerve, and that he (Smith) thought the dentist was intoxicated when he did the work.

"Drs. Sprague and Buffon both testified that they believed the deceased came to his death from the effects of the mercury used in the filling of the tooth.

"In accordance with the facts elicited, the jury returned a verdict that the deceased came to his death "by suffocation, caused by inflammation of the glands and infiltration of the tissues of the neck, producing closure of the trachea by pressure thereon; and we further believe that the above causes were brought about by the action of mercury, used in the filling of the second molar tooth of the right side of the lower jaw, by Dr. E D. Keef, of Marysville, Kansas.'"

